# Channel Plasmon Nanowire Lasers with V-Groove Cavities

**DOI:** 10.1186/s11671-018-2640-0

**Published:** 2018-07-31

**Authors:** Wei Wei, Xin Yan, Bing Shen, Jian Qin, Xia Zhang

**Affiliations:** 1 School of Mechanical and Electric Engineering, Guangzhou University, Guangzhou, 510006 China; 2 State Key Laboratory of Information Photonics and Optical Communications, Beijing University of Posts and Telecommunications, Beijing, 100876 China; 3 4catalyzer Inc., 530 Old Whitfield St, Guilford, CT 06437 USA

**Keywords:** Channel plasmon-polariton, Nanowire, Nanolaser

## Abstract

A hybrid channel plasmon nanowire laser based on GaAs/AlGaAs core-shell semiconductor nanowire and silver V-groove is proposed. The laser structure has potential capability of integrating with plasmonic waveguides, using channel plasmon-polariton modes in V-groove plasmonic waveguides. Guiding and lasing properties are numerically calculated using finite elements method. From the theoretical results, the laser could support guiding mode with a smallest diameter of 40 nm. Lasing emission could happen at a relatively low threshold around 2000 cm^− 1^ when the diameter is larger than 140 nm. A quite large Purcell factor of 180 could be achieved to enhance the spontaneous emission rate.

## Background

With cylindrical geometry and strong two-dimensional confinement of electrons, holes, and photons, independent semiconductor nanowire is ideal for semiconductor laser with reduced threshold and compact size [1–6]. Up to date, room-temperature lasing emission has been realized in ZnO, GaN, CdS, and GaAs nanowires, covering optical spectrum from ultra-violet to near-infrared [7–12]. To continue shrinking dimensions of nanowires beyond the diffraction limit, plasmonic nanowire lasers has been proposed and experimentally demonstrated, including hybrid plasmonic nanowire lasers and high-order mode plasmon nanowire lasers [13–15]. Among them, hybrid plasmonic nanowire lasers achieved much smaller dimension limit. Recently, plasmonic nanowire laser showed its capability of integrating with plasmonic waveguides, using channel plasmon-polariton (CPP) modes in V-groove plasmonic waveguides [16]. The diameters adopted in the experiment are above 300 nm. CPPs are the plasmon polaritons guided by a V-shaped groove carved in metal, which was first theoretically suggested by Maradudin and co-workers [17]. CPPs showed strong confinement, low damping, and robustness against channel bending at near-infrared wavelengths [18–20].

Here, by combining the low dissipation of hybrid plasmonic modes with the strong confinement and integration with plasmonic waveguides of CPP mode, we propose a hybrid channel plasmon nanowire (CPN) lasers and numerically investigate the modal and lasing properties. The CPN laser is comprised of a core-shell GaAs/AlGaAs nanowire and silver V-groove which is separated by an ultra-thin dielectric layer of MgF_2_, in which the diameter of nanowire locates in the range of 40 to 220 nm to explore the lasing properties beyond the diffraction limit. Due to the hexagonal shape of GaAs/AlGaAs nanowire, two integrated structures of CPN lasers will be shown in next section.

### PPN Laser Structures

**Fig. 1:**
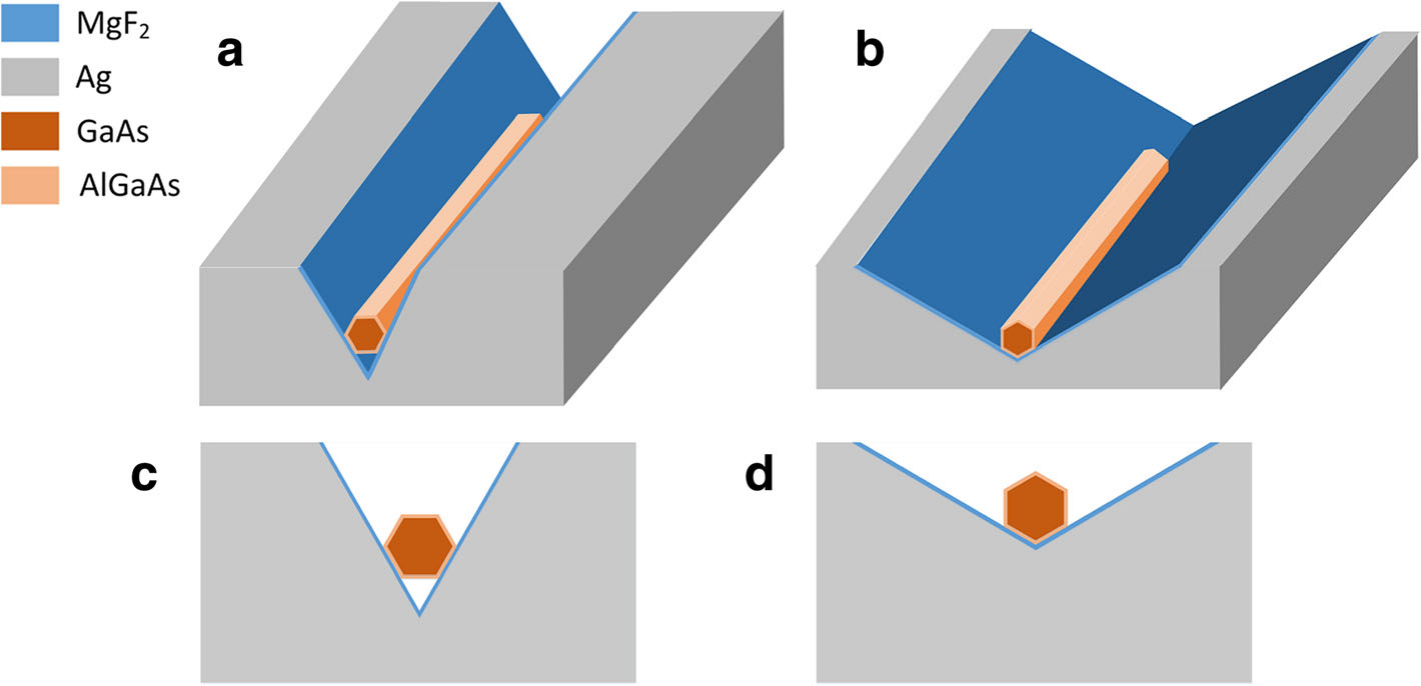
Schematic diagram of the CPN laser structures. **a** CPN-N. **b** CPN-W. **c** Cross-section of CPN-N. **d** Cross-section of CPN-W

The schematic of the CPN laser structures are demonstrated in Fig. 1, where the background material is air, the material in gray is silver, whose permittivity is described by the Drude model ${\varepsilon}_r={\varepsilon}_{\infty }-{\omega}_p^2/\left({\omega}^2+ j\gamma \omega \right)$, with *ε*_∞_=3.7, *ω*_*p*_=9.1 eV, and *γ*=0.018 eV [21]. The nanowire laying in the V-groove has a core-shell structure, the core material is GaAs and the shell material is AlGaAs. The GaAs core is passivated by a thin AlGaAs shell layer of 10 nm to improve radiative efficiency [12]. Between the nanowire and V-groove is an ultrathin dielectric layer of MgF_2_. Its thickness is fixed at 5 nm to support low-loss propagation under strong optical confinement. There are two integration ways of CPN lasers. The first one we call it CPN-N (CPN-narrow-angle) as shown in Fig. 1a, c, where the nanowire horizontally lays on the surface of V-groove with a narrow angle of 60°. The nanowire has two sides contact with dielectric layer and the V-groove surface, between the bottom side and the vertex of V-groove is air. The second one we call it CPN-W (CPN-wide-angle) as shown in Fig. 1b, d, where the nanowire vertically lays on the surface of V-groove with a wide angle of 120°. The nanowire has not only two sides contact but also a vertex contact with the dielectric layer and the V-groove surface.

**Fig. 2:**
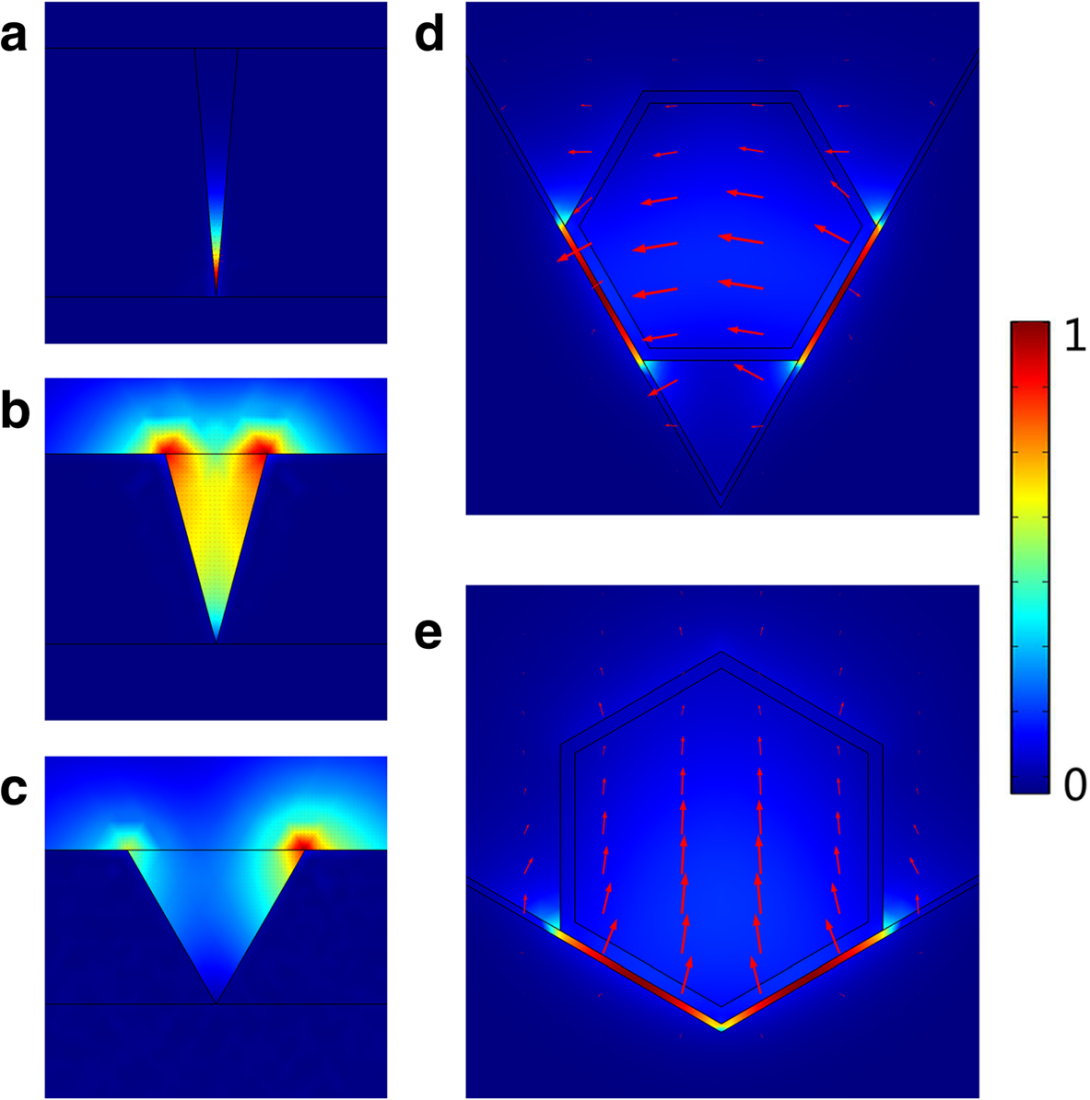
Electric field distribution in **a** groove with 10°. **b** Groove with 30°. **c** Groove with 60°. **d** CPN-N laser. **e** CPN-W laser. The red arrows indicate the orientation of electric field

Supported CPP modes in the V-groove depend on the angle and depth of the groove, especially the angle. Normally, the number of CPP modes supported by the groove decreases with the increasing angles, and in a finitely deep groove, no CPP can exist in the groove if the degree is larger than the critical degree [22]. Strong localization of CPP can be achieved in grooves with sufficiently small angles [23], which is also shown in Fig. 2. In Fig. 2a–c, the depth of groove is fixed at 1 μm, the angles of groove are 10°, 30°, and 60°, respectively. Electric field is strongly localized in the bottom of the groove with 10°, forming CPP mode. Whereas, electric field begins to distribute towards the edge of the groove with 30°, indicating the localization becomes much weaker. With the increased angle of groove to 60°, no CPP exist the groove. However, as shown in Fig. 2d, e, with the integration of nanowire, CPP still exist in wide angle of 60° and 120° (depth is smaller than 1 μm) and tightly localized inside the low-dielectric MgF_2_ layer, which is totally different from normal grooves. In a hybrid plasmonic structure like CPN cavity, the coupling between dielectric and plasmonic modes across the ultrathin dielectric layer enables ‘capacitor-like’ energy storage that allows subwavelength light propagation in non-metallic regions with nanolocalized electromagnetic field [24]. So, the electric field of CPP is strongly localized in the MgF_2_ gap between the nanowire and groove, even in the groove with wide angles. Further guiding and lasing properties in CPN-N and CPN-W lasers will be elaborated in next section.

## Results and Discussion

With the advantage of hybrid plasmonic modes, electric field can be localized in dimensions beyond the diffraction limit with low-loss propagation [25, 26]. So, our investigation focuses on the guiding and lasing properties in subwavelength diameter dimension, 40 to 220 nm. Although it is challenging to precisely control the position of nanowire with diameter below 100 nm, more or less ideal condition is considered here to explore the potential performance of CPN lasers.

**Fig. 3:**
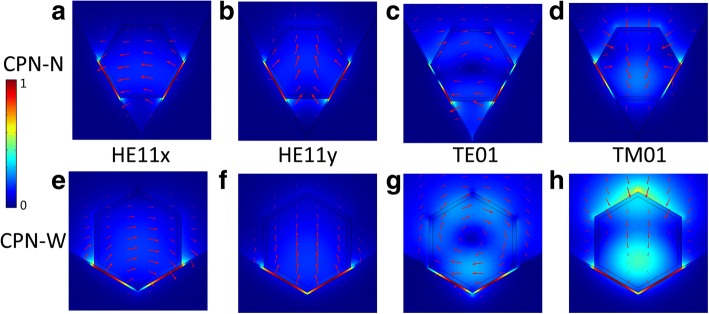
Electric field distribution of modes in CPN-N laser (**a**–**d**) and CPN-W laser (**e**–**h**). Nanowire diameter is fixed at 200 nm

Like other plasmonic nanowire lasers, more guided modes are supported in CPN lasers with the increasing diameters of nanowires. As shown in Fig. 3, the nanowire with a diameter of 200 nm incorporated in the groove can support four guided modes, HE_11x_, HE_11y_, TE_01_, and TM_01_. The surface of groove is parallel to the sides of nanowire, so the groove angle keeps invariable as the nanowire diameter changes. In a plasmonic nanowire laser with planar substrate, the nanowire has only one side contact with the substrate, leading to the coupling only between photonic modes of HE_11y_ and surface plasmons. Whereas, in a CPN structure, both HE_11x_ and HE_11y_ couple with surface plasmons forming hybrid channel plasmonic modes due to two sides contact between the nanowire and the surface of groove. For modes TE_01_ and TM_01_, electromagnetic energy inside the nanowire also couples with the surface plasmons on the groove surface forming channel plasmonic modes. The above four modes are the guided modes in CPN lasers with diameter of 200 nm, and modes cut off with the decreasing diameter.

**Fig. 4:**
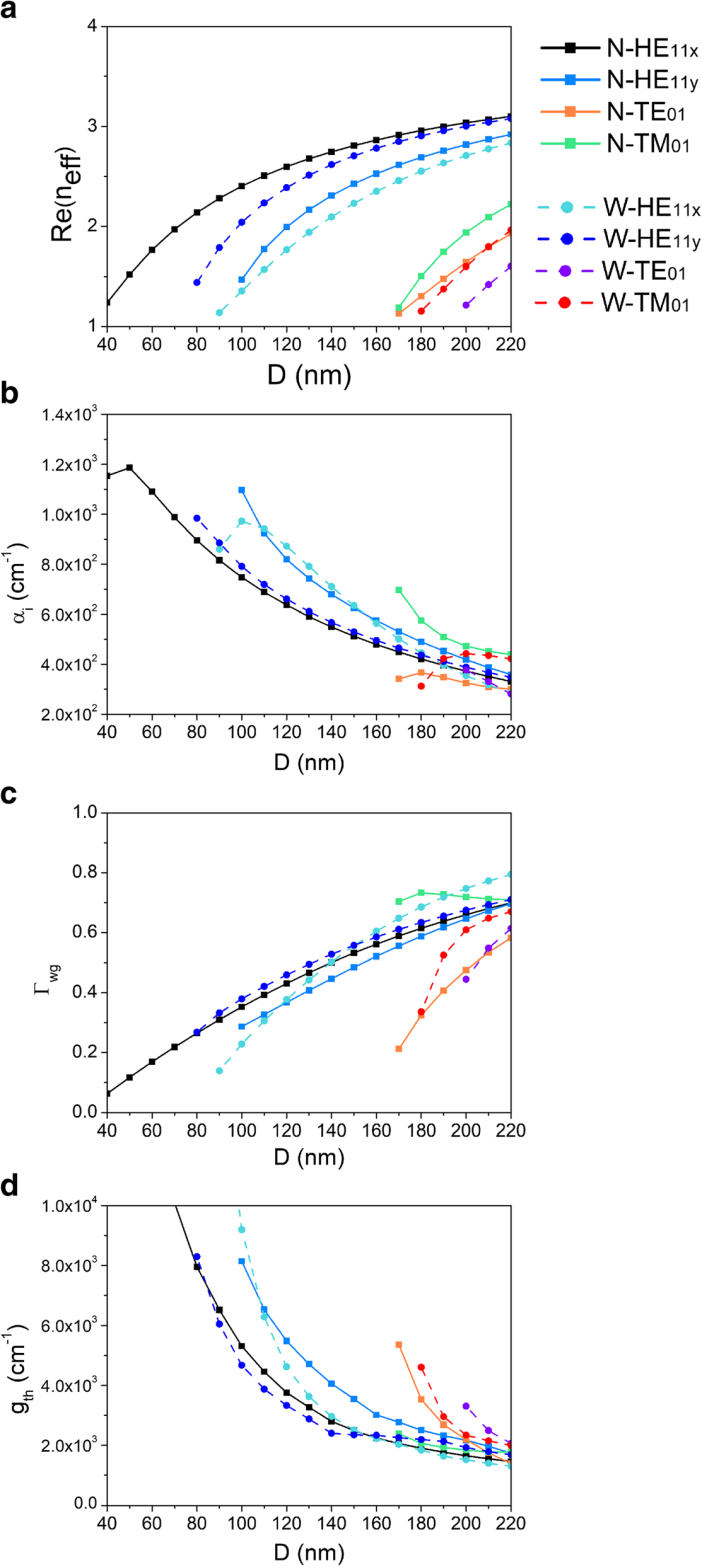
Dependences of **a** the real part of the effective index, **b** modal loss, **c** modal confinement factor, and **d** threshold gain on nanowire diameter D

To investigate the guiding and lasing properties of the CPN laser, dependences of the real part of effective index, modal loss, modal confinement factor, and threshold gain on the nanowire diameter *D* are calculated and presented in Fig. 4a–d. Modes HE_11x_, HE_11y_, TE_01_, and TM_01_ of CPN-N and CPN-W lasers are all investigated here. Properties of CPN-N and CPN-W lasers are marked as block symbol with solid line and circle symbol with dashed line, respectively. It is worth to note that the groove depth here is much larger than the nanowire diameter to eliminate the influence of the groove edge. As shown in Fig. 4a, there is a positive correlation between the real part of the effective indices Re(*n*_eff_) and nanowire diameter *D*. This behaves the same as the effective index of an individual nanowire. With the increasing diameter of nanowire, the equivalent index of the structure becomes larger, leading to the increasing modal index. As the diameter decreases, mode TE_01_ of CPN-W laser first cuts off at 200 nm, then mode TM_01_ of CPN-W laser cuts off at 180 nm, and modes TE_01_ and TM_01_ of CPN-N laser both cut off at 170 nm, whereas, the fundamental modes HE_11x_ and HE_11y_ have smaller cut-off diameters. Due to the asymmetric structure of CPN lasers, the fundamental mode no longer degenerates. Mode HE_11x_ has the smallest cut-off diameter of 40 nm during all the modes in a CPN-N laser. Mode HE_11y_ has the smallest cut-off diameter of 80 nm during all the modes in a CPN-W laser. In a CPN-N laser, Re(*n*_eff_) of mode HE_11x_ is larger than that of mode HE_11y_. Whereas, in a CPN-W laser, Re(*n*_eff_) of mode HE_11y_ is larger than that of mode HE_11x_, which results from the perpendicular component of the fundamental mode. Normally, the directions of electric field of HE_11x_ and TE_01_ are perpendicular to HE_11y_ and TM_01_, respectively. In CPN-N and CPN-W lasers, the groove angles are 60° and 120°, resulting that *x*-component of modes dominate in CPN-N lasers and *y*-component of modes dominate in CPN-W lasers, as shown in Fig. 2d, e. Thus, modes HE_11x_ and TE_01_ have larger Re(*n*_eff_) and smaller cut-off diameters in a CPN-N laser, whereas modes HE_11y_ and TM_01_ have larger Re(*n*_eff_) and smaller cut-off diameter in a CPN-W laser.

The modal loss per unit length *α*_*i*_ and modal confinement factor *Γ*_*wg*_ are significant factors of the optical cavity relevant to lasing. The modal confinement factor is an indicator of how well the mode overlaps with the gain medium, which is defined as the ration between the modal gain the material gain in the active region [27, 28]. The modal loss per unit length *α*_*i*_ can be obtained from the imaginary part of modal propagation constant *k*_*z*_ as *α*_*i*_ = 2 Im[*k*_*z*_]. As shown in Fig. 4b, the modal loss of CPN-N and CPN-W lasers behaves negatively correlated with the nanowire diameter *D*. Whereas as shown in Fig. 4c, the confinement factor of CPN-N and CPN-W lasers behaves positively correlated with the nanowire diameter *D*. With the decreasing diameter of nanowire, the electromagnetic energy cannot be localized well inside the nanowire, more and more electromagnetic energy leaks. Part of electromagnetic energy scatters outside from the upper part of nanowire, and part of energy interacts with groove surface leading to more metal dissipation. It is interesting to note that mode TM_01_ in CPN-N laser has both relatively large confinement factor and modal loss. This can be attributed to the distribution of electric field of mode TM_01_. As shown in Fig. 3d, electromagnetic energy distributes both inside the nanowire and around its surface. Though the confinement is tighter, the electromagnetic energy has stronger interaction with the metal groove. Importantly in Fig. 4c, as the nanowire diameter increases, the confinement factor becomes larger, indicating that the electromagnetic energy is confined in the cavity and overlaps well with the active region and potentially lower the lasing threshold.

Lasing threshold is the lowest excitation level at which laser output is dominated by stimulated emission rather than spontaneous emission. The threshold gain *g*_th_, which describes the required gain per unit length for lasing, is defined as ${g}_{\mathrm{th}}=\frac{1}{\varGamma_{wg}}\left[{\alpha}_i+\frac{1}{L}\ln \left(\frac{1}{R}\right)\right]$, where *R* denotes the geometric mean of the reflectivity of the end facets of nanowire and *L* is the length of the nanowire F-P cavity [29]. The length *L* is fixed at 10 μm, which fits the experimental data in Ref. [12]. It needs to be noted that the nanowire here is the same as Ref. [11, 12], in which grown method of Au-particle catalyst was adopted. So, there is a gold cap on the top of nanowire. For the end facet with a gold cap, the reflectivity is larger than the other end facet, reaching around and more than 70%. We depict dependence of threshold gain *g*_*th*_ on *D* in Fig. 4d. It is obvious that the threshold gain decreases with the increasing nanowire diameter. This accords with the behaviors of modal loss and confinement factor, which are key factors of threshold gain. As the nanowire diameter increases, the electromagnetic energy is confined better inside the nanowire, leading to larger confinement factor and smaller energy leakage loss. Thus, the threshold gain becomes lower. In smaller diameter range, the threshold gain of mode HE_11x_ is lower than mode HE_11y_ in CPN-N laser, the threshold gain of mode HE_11y_ is lower than mode HE_11x_ in CPN-W laser. This also proves the mode HE_11x_ and HE_11y_ revolves in CPN lasers, due to the effect of groove angles on the electric field components.

**Fig. 5:**
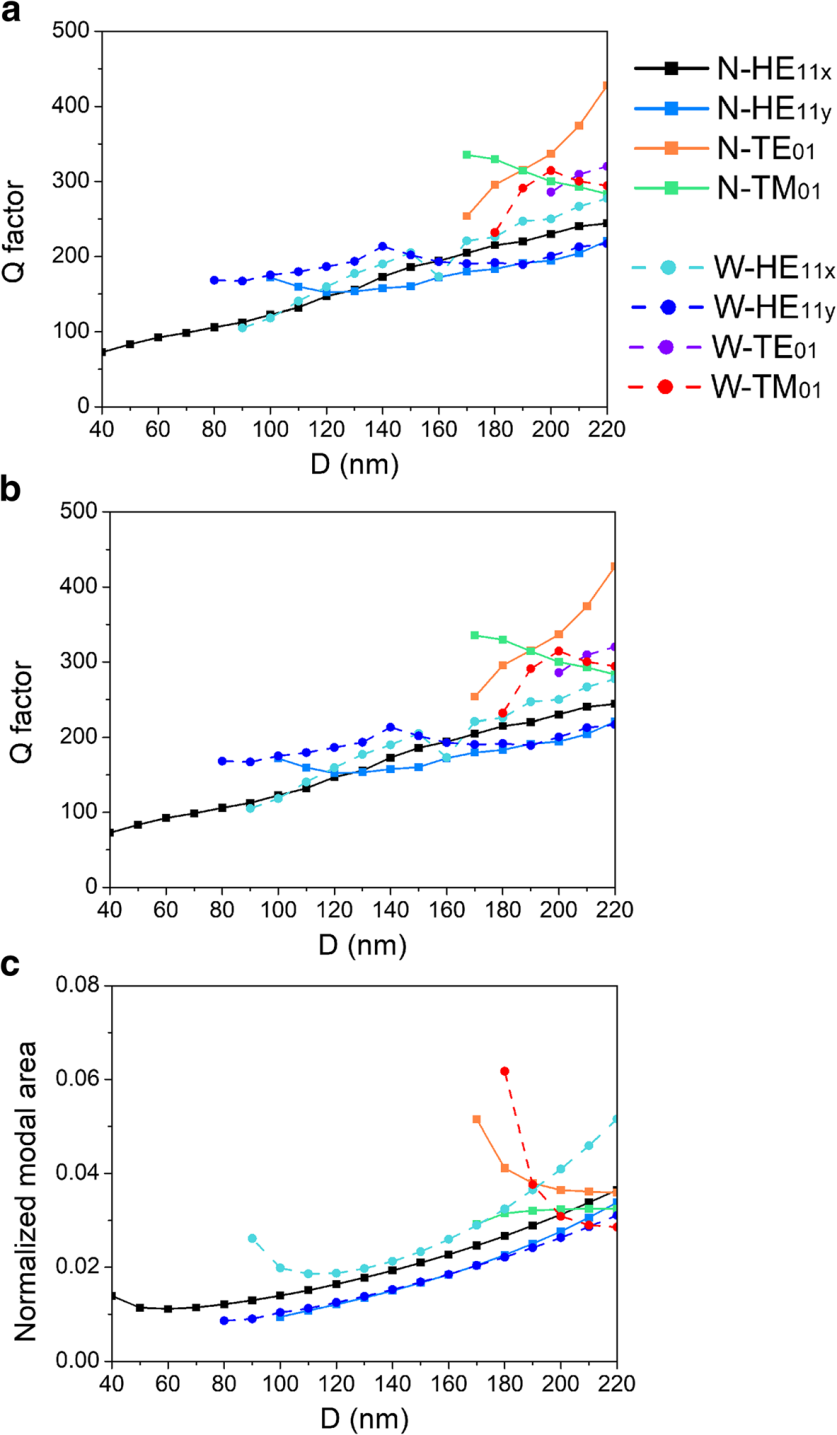
Dependences of **a** quality factor, **b** Purcell factor, and **c** normalized modal area on nanowire diameter *D*

Quality factor *Q* of a cavity mode is indicative of how long the stored energy of that mode remains in the cavity when interband transitions are absent, which is related to the photon lifetime *τ*_*p*_ enters the rate equation via the resonance frequency ω of the mode. For a F-P cavity, the quality factor is defined in the methods section [30]. High quality factor indicates a low rate of energy loss relative to the stored energy of the cavity and the oscillations die out slowly. So, the device can lase at a lower threshold and hence pump power could be reduced. We depict *Q* factor as functions of *D* in Fig. 5a. There are positive correlations between quality factors of all modes and diameter *D*, except for modes TM_01_ in CPN-N and CPN-W lasers. This could be attributed to the electric field distribution of mode TM_01_, which has been discussed in the above. Furthermore, spontaneous emission rate in a nanolaser like CPN laser partly depends on environment of a light source. According to Fermi’s golden role, the spontaneous emission rate of an emitter is proportional to the local density of optical states (LDOS) [31]. In an environment that structure is at the scale of the wavelength, the LDOS can be spatially controlled [32]. As a result, the LDOS of an emitter can be locally increased together with the rate of spontaneous emission or decreased by the subwavelength microcavity, which is called the Purcell effect [33]. The nanolocalized electromagnetic energy can decrease the lasing threshold by enhancing the spontaneous emission rate via the Purcell effect. In CPN-N and CPN-W lasers, electromagnetic energy is tightly localized at subwavelength scale, resulting in large Purcell factors as shown in Fig. 5b. The metal groove modifies the dielectric environment around the nanowire and constructs a subwavelength cavity, enabling an ultra-small volume and coupling between an exciton and a microcavity mode. With the decreasing diameter, the Purcell factor increases sharply and reaches more than 100. Moreover, a large LDOS can enhance not only the rate of spontaneous emission, but also stimulated emission process in the lasing action. Lasing action could be easier achieved because the nanolocalized electromagnetic field of the hybrid plasmonic mode not only makes the excitons in the nanolaser diffuse rapidly towards areas of faster recombination improving the overlap between material gain and plasmonic mode but also stimulates excited-state particles to transfer energy into plasmons of the same frequency, phase, and polarization. To quantify the subwavelength localization scale, the normalized modal area calculated using method in Ref. [13] and presented in Fig. 5c. Compared to Fig. 5b, the Purcell factor is inversely proportional to the normalized modal area, which proves that the cavity at subwavelength scale increases the Purcell factor and therefore enhances the spontaneous emission rate.

## Conclusions

We proposed a CPN laser structure based on semiconductor nanowire and metal V-groove together with an ultrathin layer of dielectric. With the presence of high-index nanowire, channel plasmons can exist in the grooves with relatively large angles forming hybrid channel plasmonic modes. The metal groove modifies the dielectric environment around the nanowire and constructs a subwavelength cavity enabling the enhancement of spontaneous emission rate. Guiding and lasing properties were investigated using finite elements method. The fundamental mode HE_11x_ in CPN-N laser has a very small cut-off diameter, enabling ultra-small footprint of on-chip lasers. With the advantage of high confinement and ultra-small normalized modal area, the Purcell factor can reach more than 150 to greatly enhance the spontaneous emission rate. Besides, this CPN laser also has potential capability of integrating with plasmonic waveguides using CPP modes in V-groove plasmonic waveguides, which would find important applications in on-chip optical interconnections.

## Methods/Experimental

Guiding and lasing properties were numerically calculated using finite elements method with the scattering boundary condition in the frequency, which is a commonly employed approach to mimic the necessary open boundary. The electric field distributions of the eigenmodes of CPN lasers are directly obtained by mode analyses. The guiding properties are calculated by the complex propagating constant with *β + iα*. The real part of the modal effective index is calculated by *n*_eff_ = Re(*n*_eff_) = *β*/*k*_*0*_, where *k*_*0*_ is the vacuum wavevector. The effective mode area is calculated using [24]

1 $$ {A}_m=\frac{W_m}{\max \left\{W(r)\right\}}=\frac{1}{\max \left\{W(r)\right\}}{\iint}_{\infty }W(r){d}^2r $$

where *W*_*m*_ is the total mode energy and *W(r)* is the energy density (per unit length flowed along the direction of propagation). For dispersive and lossy materials, the *W(r)* inside can be calculated using Eq. (2):

2 $$ W(r)=\frac{1}{2}\left(\frac{d\left(\varepsilon (r)\omega \right)}{d\omega}{\left|E(r)\right|}^2+{\mu}_0{\left|H(r)\right|}^2\right) $$

Quality factor and Purcell are defined as [30, 33]

3 $$ \kern0.75em \frac{1}{Q}=\frac{1}{{\omega \tau}_p}=\frac{\nu_{g,z}\left(\omega \right)}{\omega}\left[{\alpha}_i+\frac{1}{L}\ln \left(\frac{1}{R}\right)\right] $$

4 $$ {F}_p=\frac{3}{4{\pi}^2}{\left(\frac{\lambda }{n}\right)}^3\left(\frac{Q}{V_{eff}}\right) $$

Equations to calculate modal loss, modal confinement factor, and threshold gain are provided in the main text; we do not narrate here again.

## Abbreviations

CPN: Channel plasmon nanowire
CPN-N: Channel plasmon nanowire-narrow-angle
CPN-W: Channel plasmon nanowire-wide-angle
CPP: Channel plasmon-polariton
